# Linking teacher support to achievement emotion profile: the mediating role of basic psychological need satisfaction

**DOI:** 10.3389/fpsyg.2024.1352337

**Published:** 2024-08-01

**Authors:** Yang Yang, Shaoying Gong, Yang Cao, Yin Qiu, Xizheng Xu, Yanqing Wang

**Affiliations:** ^1^Key Laboratory of Human Development and Mental Health of Hubei Province, School of Psychology, Central China Normal University, Wuhan, China; ^2^Mental Health Education Center, Huanghuai University, Zhumadian, China; ^3^School of Health Humanities, Hubei Shizhen Laboratory, Hubei University of Chinese Medicine, Wuhan, China; ^4^Wuhan College, Wuhan, China; ^5^Hunan Police Academy, Changsha, China

**Keywords:** emotion co-occurrence, teacher support, basic psychological need satisfaction, three-step latent profile analysis, achievement emotion

## Abstract

The current study used a person-centered approach to explore the co-occurrence of college students’ achievement emotions. It also examined the impact of teacher support on achievement emotion profiles and the mediating effect of need satisfaction. A total of 866 college students participated in the survey. A robust three-step latent profile analysis was employed to analyze the data. Four profiles of achievement emotions were identified: moderate mixed emotions, the blends of high positive emotions, the blends of moderate positive emotions, and high mixed emotions. Higher perceived teacher support was associated with a greater likelihood of being classified into the blends of moderate positive emotion profile or the blends of high positive emotion profile. Moreover, basic psychological need satisfaction mediated the relationship between teacher support and the four emotion profiles. Our findings contribute to a more comprehensive understanding of the role of teacher support in shaping achievement emotion profiles, helping to broaden the application of self-determination theory to explain the mechanism by which external support influences emotion profiles.

## 1 Introduction

Achievement emotions have received growing attention from researchers and practitioners of education due to the increasing recognition of their importance in learning. Achievement emotions, defined as those related to achievement activities or achievement outcomes (Pekrun, 2006), influence the learning process, including motivation, strategy use, engagement, and performance (Pekrun et al., 2023). Over the last 20 years, researchers have mostly explored achievement emotions from a variable-oriented perspective, assessing the relationship between discrete achievement emotions and their antecedents and learning outcomes (Pekrun and Linnenbrink-Garcia, 2014). Excessive emphasis on discrete achievement emotions ignores the interplay between these emotions. In fact, learners typically experience multiple emotions simultaneously during learning, a phenomenon referred to as “emotion co-occurrence” (Moeller et al., 2018; Kuklick and Lindner, 2023). Previous studies have found that the co-occurrence of multiple emotions significantly predicted learning performance (Robinson et al., 2020; Tze et al., 2020; Karamarkovich and Rutherford, 2021; Earl et al., 2024). However, investigations into the occurrence of achievement emotions in higher education have primarily emphasized a narrow range of cultural contexts, predominantly within Western cultures. As a result, research on Chinese university students is relatively scarce. Furthermore, there has been limited research exploring how environmental factors (e.g., teacher support) influence the occurrence of emotions. Therefore, the study aims to identify the co-occurrence patterns of achievement emotions among Chinese college students, examine the antecedent factors associated with these patterns, and elucidate the mechanisms contributing to the formation of distinct emotion patterns.

### 1.1 Co-occurrence of multiple achievement emotions

In everyday life, individuals frequently experience the co-occurrence of multiple emotions. Watson and Stanton (2017) have identified four distinct patterns of emotion co-occurrence: non-emotional, pure emotion, emotion blends, and mixed emotions. The non-emotional pattern emphasizes the absence of any obvious emotional experience. The pure emotion pattern refers to individuals mainly experiencing a single discrete emotion, emphasizing the importance of a singular emotion. The emotion blends pattern refers to the simultaneous feeling of emotions with similar valence, emphasizing the combination of emotions with the same valence. The mixed emotions pattern refers to the concurrent feeling of various emotions with different valences, emphasizing the combination of emotions with different valences (Larsen and McGraw, 2011).

This classification of emotion patterns is not only applicable to daily life but also to learning situations. Research on the co-occurrence of achievement emotions has focused on identifying patterns or profiles of emotions using person-centered approaches such as clustering, latent profile analysis, and latent category analysis. Only a few studies have identified common emotion profiles in college students’ learning (Jarrell et al., 2016, 2017; Postareff et al., 2017; Robinson et al., 2017, 2020; Tze et al., 2020; Earl et al., 2024). For example, Postareff et al. (2017) found that freshmen in Finland during the transition to university experienced three emotion profiles: two blends of positive emotions and one blends of negative emotions. Robinson et al. (2017) explored achievement emotion profiles of American college students studying science and identified two emotion profiles: emotion blends and mixed emotions. Tze et al. (2020) identified three profiles among Canadian university students: blends of positive emotions, blends of negative emotions, and mixed emotions. Additionally, Cheng et al. (2023) identified three achievement emotion profiles among vocational college students during online learning: blends of negative emotions, non-emotional, and pure positive emotions. From prior studies, we found that the emotion profiles of college students exhibit remarkable diversity. Typically, there are two to three emotion profiles, consistently including the blends of positive emotion profile and the blends of negative emotion profile. It is worth noting that most prior research has been primarily rooted in Western cultures, overlooking the potential impact of Eastern cultures (e.g., Chinese culture) on achievement emotion profiles. The use of a person-centered approach to identify emotion profiles is contingent upon the levels of emotions. The mean levels of achievement emotions varied across different cultures. In Chinese culture, academic achievement holds significant value, as it is often linked to family status and social reputation (Ryan and Deci, 2017), thereby influencing the levels of students’ achievement emotions. For example, Frenzel et al. (2007) found that Chinese students reported more enjoyment, pride, anxiety, shame, and less anger compared with German students. Additionally, a meta-analysis found that, in collectivist cultures, game-based learning has a greater impact on students’ negative academic emotions (Lei et al., 2022). We speculate that there may be cultural differences in achievement emotion profiles. Therefore, the first aim of this study is to explore the number and characteristics of possible achievement emotion profiles among Chinese college students during daily learning.

Existing studies have suggested that individuals with different emotion profiles exhibit significant differences in action orientation (Fernando et al., 2014) and academic achievement (Ganotice et al., 2016; Tze et al., 2020). Specifically, individuals in the blends of positive emotion profile tended to have better adaptive outcomes compared to those in the blends of negative emotion profile (Ganotice et al., 2016; Tze et al., 2020; Earl et al., 2024). However, the effect of the mixed emotion profile on academic achievement is inconsistent. One study found that students in the mixed emotion profile performed similarly to students in the blends of positive emotion profile and performed better than individuals in the blends of negative emotion profile (Robinson et al., 2017). Another study found that, compared to the blends of positive emotion profile, the mixed emotion profile was a negative predictor of American College Testing scores (Robinson et al., 2020). In addition, in a longitudinal study, university students in the stable mixed profile performed worse than those in the stable positive emotion profile (Tze et al., 2020). Despite inconsistent results, emotion profiles could have a significant impact on academic achievement. Therefore, it is crucial to explore the factors that contribute to students’ emotion profiles.

### 1.2 Antecedents that influence the co-occurrence of achievement emotions

Which factors can predict distinct achievement emotion profiles? Previous research has predominantly focused on exploring the determinants of learners’ emotion profiles within the framework of the control-value theory (CVT). Notably, motivational appraisals (i.e., control and value) exert a significant influence on students’ emotion profiles (Robinson et al., 2017, 2020; Karamarkovich and Rutherford, 2021; Cheng et al., 2023). Individuals with high subjective evaluations of control and value of tasks are more likely to belong to the blends of positive emotion profile, while those with low subjective evaluations are more likely to belong to the blends of negative emotion profile. Individuals with moderate subjective evaluations of control are likely to belong to the mixed emotion profile. However, insufficient attention has been paid to the role of external contextual factors. As is commonly known, teacher support is an important environmental factor that affects students’ achievement emotions (Ryan and Deci, 2000; Pekrun, 2006). Does teacher support influence emotional profiles, and if so, how? Exploring this question could help us understand the role and pathways of teacher support in influencing the co-occurrence of achievement emotions.

#### 1.2.1 Teacher support as an antecedent

From the perspective of Basic Psychological Needs Theory (BPNT), a core mini-theory within the framework of Self-Determination Theory, teacher support is defined as a social need-relevant context in this study, which is a multidimensional construct including autonomy support, competence support, and emotional support provided by teachers (Deci and Ryan, 2000; Skinner et al., 2008). Autonomy support refers to teachers providing students with choices to pursue their interests, offering opportunities for self-initiative, encouraging free expression, and minimizing the use of controlling language (Deci and Ryan, 2000; Su and Reeve, 2011). Competence support refers to the academic support provided by teachers, such as instructional assistance, guidance, and informational feedback (Federici and Skaalvik, 2013). Emotional support refers to the warmth and emotional connection perceived by students in their relationship with teachers, which is fostered through teachers’ care and affection (Patrick et al., 2007; Song et al., 2015). According to BPNT and CVT, teacher support is an important social factor that influences students’ achievement emotions (Ryan and Deci, 2000; Pekrun, 2006). When students perceive support from teachers regarding their autonomy, they are more likely to demonstrate more engagement in learning (Olivier et al., 2021) and experience heightened intrinsic motivation (Pap et al., 2021), leading to increased positive emotions (Liu et al., 2021). When students perceive teachers’ support for their competence, they are more likely to gain academic self-efficacy to handle learning tasks (Affuso et al., 2023), thereby fostering more positive achievement emotions and less negative achievement emotions (Wang et al., 2022). Meanwhile, when students receive emotional support from teachers, they feel understood and cared for, fostering emotional security and relaxation. This experience, in turn, can lead to students experiencing more positive emotions.

Some research studies have also confirmed that teacher support can directly influence students’ emotions in the academic setting (Pekrun, 2006; Reeve, 2012). Liu et al. (2021) found that teacher support was significantly positively correlated with students’ enjoyment and relaxation, negatively correlated with anxiety. Hejazi and Sadoughi (2023) demonstrated that teacher support enhanced the enjoyment of foreign language learners. A meta-analysis categorized discrete achievement emotions into positive and negative achievement emotions and found a moderate positive correlation between teacher support and students’ positive achievement emotions and a moderate negative correlation with negative achievement emotions (Lei et al., 2018). However, there has been no research exploring the impact of teacher support on students’ emotion profiles from the perspective of multiple emotions, so the second aim of this study is to investigate whether teacher support affects the emotion profiles of college students.

#### 1.2.2 Need satisfaction as a mediator

According to BPNT, individuals generally have three fundamental needs: autonomy, competence, and relatedness. These needs are broadly defined as essential resources that individuals naturally seek to enhance self-organization, adaptation, and prosperity (Deci and Ryan, 2000; Vansteenkiste et al., 2020). Basic psychological needs act as the mechanism linking need-relevant conditions with outcomes (Vansteenkiste et al., 2020). From this perspective, the influence of teacher support on students’ achievement emotion profiles may be mediated by the satisfaction of their basic psychological need satisfaction.

On the one hand, both theoretical and empirical studies have shown that need satisfaction is largely influenced by a need-relevant context (Deci and Ryan, 2008; Vansteenkiste et al., 2020). Teacher support plays a crucial role in promoting need satisfaction (Skinner et al., 2008). When teachers provide students with choice or respect, students’ autonomy needs are more likely to be met. When teachers clearly express their expectations and give academic support, students might also feel competent, thus satisfying their need for competence. When teachers demonstrate enthusiasm, love, understanding, or reliability toward their students, students’ need for relatedness is more likely to be fulfilled. Previous research has shown that teacher support was significantly positively related to need satisfaction for elementary school students (Zhou et al., 2019; Ahn et al., 2021) and upper secondary school students (Jeno and Diseth, 2014). Furthermore, a meta-analysis study found that the teachers’ autonomous support positively predicted students’ need satisfaction (Bureau et al., 2022).

On the other hand, the fundamental assumption of BPNT suggests that the satisfaction of basic psychological needs is necessary for thriving and well-being (Ryan and Deci, 2017), as evidenced by the fostering of positive emotions and the attenuating of negative emotions (Deci and Ryan, 2002). Cross-sectional studies indicated a close correlation between satisfaction of basic psychological needs and emotions (Flunger et al., 2013; Tóth-Király et al., 2020; Wang, 2024). For example, Wang (2024) surveyed 391 Chinese undergraduates, revealing that basic psychological need satisfaction was positively associated with positive achievement emotions while studying English. Meta-analyses across various domains investigating the consequences of basic psychological need satisfaction have consistently shown a positive correlation between need satisfaction and positive emotions, as well as a negative correlation with negative emotions, across a diverse array of populations, including older (Tang et al., 2020) patients (Schutte and Malouff, 2021). Additionally, a meta-analysis dedicated to exploring the relationship between need satisfaction and emotions demonstrated that a higher level of basic need satisfaction is associated with a higher level of positive emotions and a lower level of negative emotions (Stanley et al., 2021). Longitudinal studies have also shown that frustration, whether occurring during the ordinary academic year or the COVID-19 pandemic, could predict university students’ future experiences of negative emotions (Levine et al., 2022). Furthermore, teachers’ autonomous support had an indirect effect on achievement emotions (e.g., hope) through need satisfaction among college students (Bordbar, 2021). Based on the above theory and prior studies, need satisfaction could mediate the association between teacher support and discrete achievement emotions. However, previous studies have not focused on the role of need satisfaction in how teacher support influences the co-occurrence of students’ achievement emotions. Expanding to the perspective of multiple emotions, it is speculated that individuals whose psychological needs are met may experience multiple positive emotions simultaneously and are more likely to be in the blends of positive emotion profile. Conversely, if individuals’ needs are not met, they are more likely to experience a variety of negative emotions and belong to the blends of negative emotion profile. Does need satisfaction play a mediating role in the relationship between teacher support and emotion profiles? The third aim of this study is to explore the mediating effect of need satisfaction.

### 1.3 Current study

Variable-centered research methods cannot answer questions about the co-occurrence of achievement emotions. However, a person-centered approach is a useful tool to identify trends (i.e., profiles) in the co-occurrence of achievement emotions (Jarrell et al., 2016). To date, there is a relative scarcity of research, particularly with regard to Chinese college students. There are few studies that examine the correlation between teacher support and students’ achievement emotion profiles, as well as whether this relation is mediated by need satisfaction. Therefore, this study aims not only to use latent profile analysis to identify distinct profiles of achievement emotions among Chinese college students but also to explore the impact and underlying mechanisms of predictors on these profiles. We mainly address three questions.

**Research question (RQ_1_):** How many emotion profiles would exist among Chinese college students? We focus on four typical emotions: enjoyment, anger, boredom, and hope. Enjoyment, anger, and boredom occur most frequently during learning and are best documented in the literature (Camacho-Morles et al., 2021). According to Cheng et al. (2023), it is advisable to balance the ratio of positive and negative emotions. Therefore, we have chosen a positive emotion, such as hope, which is believed to be linked to a sense of control. Based on inconsistent findings, we are unable to predict the specific existence of achievement emotion profiles, so we have made a speculation that Chinese college students might exhibit different achievement emotion profiles (H_1_).

**RQ_2_:** How would antecedents affect students’ achievement emotion profile? Existing studies have found the impact of teacher support on students’ discrete achievement emotions. However, there has been no research exploring the impact of teacher support on students’ emotion profiles. A meta-analysis has found that higher levels of teacher support are associated with more positive emotions and fewer negative emotions (Stanley et al., 2021). We speculate that higher levels of teacher support may make it more likely for a student to belong to the blends of positive emotion profile and less likely to belong to the blends of negative emotion profile. Additionally, previous research has shown that female and male students tend to report different levels of achievement emotions (Pekrun et al., 2011). Furthermore, prior achievement has been shown to predict emotion profiles (Karamarkovich and Rutherford, 2021). Given the current lack of sufficient research to explore the specific relationship between different antecedent variables and achievement emotion profiles, this study proposes a general hypothesis: antecedents (gender, prior achievement, teacher support) might predict different emotion profiles (H_2_).

**RQ_3_:** Does need satisfaction mediate the relationship between teacher support and membership in different achievement emotion profiles? According to BPNT, basic psychological needs are the mechanism linking psychological need-relevant conditions with outcomes (Vansteenkiste et al., 2020). We hypothesize that need satisfaction might mediate the relationship between teacher support and membership in different achievement emotion profiles (H_3_).

## 2 Methods

### 2.1 Participants and procedure

A total of 913 college students from two universities in central China participated in the survey. We excluded data from students whose effective time of questionnaire filling was either too short or too long (27, e.g., shorter than 3 min or longer than 30 min) and those who provided invalid responses (20, e.g., participants responded with the same pattern like “1, 2, 3, 4, 5, ……” or “1, 1, 1, 1, 1, ……”). A total of 866 valid questionnaires were obtained. Among the 866 valid responses from students, 324 were female, accounting for 37.41% of the participants, and 542 were male, accounting for 62.59% of the participants. A total of 610 freshmen accounted for 70.44%, while 256 sophomores and juniors accounted for 29.56%. Additionally, there were 379 students majoring in the humanities, comprising 43.76%, and 487 students majoring in science and engineering, comprising 56.24%. Furthermore, 272 students were from urban areas, and 594 students were from rural areas. The study was approved by our institution’s research ethics committee. Before filling out the questionnaire, participants were informed of the purpose of the study. All participants participated voluntarily. The main purpose of the research was explained in detail when the questionnaire was distributed. The participants were asked to complete a series of questionnaires, including demographic information and items assessing teacher support, need satisfaction, and achievement emotions. All the questionnaires were in Chinese.

### 2.2 Measures

**Teacher support:** The social context questionnaire (Belmont et al., 1988), which was compiled in Chinese, was used to assess the level of teacher support (Cheng, 2020). The questionnaire included three types of teacher support: autonomous support (seven items; a sample item is “My teacher allows me to arrange my own learning freely”), structured support (six items; a sample item is “My teacher will check my preparation before talking about new content”), and emotional support (eight items, a sample item is “My teacher knows me well”). The questionnaire adopted the 5-point Likert scale (1 = Strongly Disagree, 5 = Strongly Agree), and some items were scored reversely. Cronbach’s α for the current study was 0.88.

**Achievement emotions:** The short version of the Achievement Emotions Questionnaire (AEQ) was adapted to measure students’ class-related emotions (Bieleke et al., 2021). Enjoyment and hope were represented as positive emotions, while anger and boredom were represented as negative emotions. Each emotion was evaluated through three items (A sample item is “I enjoy the challenge of learning the material”) assessed using the 5-point Likert scale (1 = Strongly Disagree, 5 = Strongly Agree). Cronbach’s α for the current study of four emotions were 0.89, 0.93, 0.93, and 0.97, respectively.

**Need satisfaction:** Participants completed the Balanced Measure of Psychological Needs Questionnaire (Sheldon and Hilpert, 2012) to assess autonomy, competence, and relatedness need satisfaction. The scale consists of nine items (A sample item is “I do well in this class, even on the hard things”). The scale was assessed using the 5-point Likert scale (1 = strongly disagree, 5 = strongly agree). Cronbach’s α for the current study was 0.95.

**Prior achievement:** Due to the diverse majors represented among the students in our study, we asked students for subjective evaluations of their achievement. We used one item, “Your prior achievement in the last term is _______,” with the responses given a grade ranging from 1 (poor) to 4 (excellent).

### 2.3 Data analysis

To better answer RQ1 and RQ2, we utilized a three-step latent profile analysis to identify subgroups of achievement emotion and explore the associations between these subgroups and predictors. The three-step latent profile analysis has several advantages, including not having to re-calculate estimations of the latent profile analysis when including auxiliary variables or distal outcomes while also taking account of a classification uncertainty rate (Asparouhov and Muthén, 2014; Collier and Leite, 2017). To identify the potential profiles of achievement emotions among college students, a number of fit indices were used, such as the Akaike information criterion (AIC), the Bayesian information criterion (BIC), the sample size adjusted BIC (ABIC), the Vuong-Lo–Mendell–Rubin adjusted likelihood ratio test (VLMR-LRT), the bootstrap likelihood ratio test (BLRT), and the entropy. Other variables, such as gender, prior achievement, and teacher support, were treated as auxiliary variables (Asparouhov and Muthén, 2014). Mplus 8.0 was utilized to analyze the models, with the R3STEP command employed for predicting membership in a latent class model. Logistic regression coefficients are presented as odds ratios (ORs) with 95% confidence intervals (95% CIs).

To answer RQ3, instead of a multinomial profile variable that compares each profile to a reference profile, we used profile membership as a binary variable (Karamarkovich and Rutherford, 2021). We explored the possible mediating role of need satisfaction on the relationship between teacher support and achievement emotion profiles through a mediation analysis using the Lavaan R package. We conducted a comprehensive analysis of total, direct, and indirect effects using 1,000 bootstraps to obtain bias-corrected 95% CIs.

## 3 Results

### 3.1 Preliminary analyses

Table 1 presents descriptive statistics and bivariate correlations. There was a significant correlation among all the variables. Teacher support was positively correlated with positive emotions, and need satisfaction was negatively correlated with negative emotions. Need satisfaction was positively correlated with positive emotions and negatively correlated with negative emotions.

**Table 1:** Descriptive statistics and bivariate correlations.

Variables	M ± SD	Skew	Kurtosis	1	2	3	4	5	6
1. Prior achievement	2.97 ± 0.92	−0.57	−0.54	1					
2. Teacher support	3.54 ± 0.51	0.68	−0.32	0.07	1				
3. Need satisfaction	5.18 ± 0.97	0.31	−0.65	0.11^**^	0.43^**^	1			
4. Enjoyment	3.95 ± 0.61	0.10	−0.34	0.14^**^	0.49^**^	0.63^**^	1		
5. Hope	3.92 ± 0.63	0.10	−0.22	0.14^**^	0.46^**^	0.64^**^	0.83^**^	1	
6. Anger	2.42 ± 1.05	0.39	−0.61	−0.09^**^	−0.62^**^	−0.08^*^	−0.25^**^	−0.23^**^	1
7. Boredom	2.33 ± 1.10	0.43	−0.71	−0.14^**^	−0.61^**^	−0.07^*^	−0.27^**^	−0.24^**^	0.90^**^

^*^p < 0.05, ^**^p < 0.01.

### 3.2 Profiles of achievement emotions

**Figure 1:**
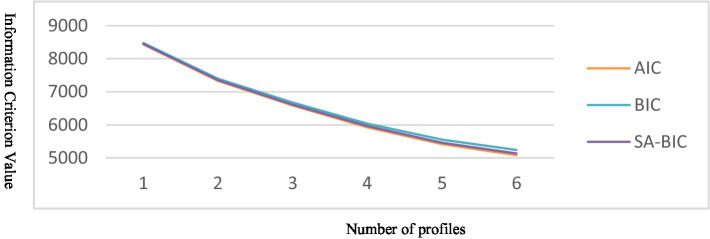
Plot of information criterion values.

The results of the profile analysis are shown in Tables 2, 3. The four-profile model was chosen as the optimal solution for the following reasons: First, the four-profile solution produced a superior fit than the two- and three-profile solutions according to the following five indices (AIC, BIC, ABIC, VLMR-LRT, and BLRT). The entropy of the four-profile model was 0.96, which was typically high (Clark and Muthén, 2009). Using the sample size adjusted BIC (Figure 1), the four-profile model exceeded the “elbow,” suggesting that any further decrease in the indices (AIC, BIC, ABIC) would lead to diminishing returns in terms of improving model fit (Nylund-Gibson et al., 2023). Second, although the fit indices for the five six-profile models were somewhat better than those for the four-profile model, each had a profile comprising less than 5% of the sample, which led to their rejection (Nylund-Gibson et al., 2023). Third, the values on the main diagonal of the matrix’s four-profile model were discovered to be near 1 (Table 3), suggesting great classification accuracy. Finally, the results of ANOVA showed that there were significant differences in four discrete emotions among four profiles (Table 4): enjoyment (*F* [3, 862] = 195.10; *p* < 0.001;η^2^ = 0.40); hope (F [3, 862] = 180.41; *p* < 0.001; η^2^ = 0.39); anger (F [3, 862] = 1393.50; *p* < 0.001;η^2^ = 0.83); and boredom (F [3, 862] = 4502.05; *p* < 0.001;η^2^ = 0.94).

**Table 2:** Indices for latent profile analysis.

No. of groups						
	1	2	3	4	5	6
K	8	13	18	23	28	33
AIC	8,438	7,331	6,588	6,598	5,418	5,083
BIC	8,476	7,393	6,673	6,707	5,551	5,240
SA-BIC	8,450	7,352	6,616	6,634	5,462	5,135
VLMR-LRT		1116^**^	753^**^	652^**^	869^**^	335^**^
BLRT		1084^**^	732^**^	671^**^	844^**^	326^**^
Entropy		0.89	0.89	0.96	0.97	0.95
*n* in each profile		C1 = 377 C2 = 489	C1 = 292 C2 = 442 C3 = 132	C1 = 220 C2 = 250 C3 = 268 C4 = 128	C1 = 210 C2 = 266 C3 = 251 C4 = 116 C5 = 23	C1 = 164 C2 = 261 C3 = 141 C4 = 124 C5 = 153 C6 = 23
smallest profile (%)		43.53	15.24	14.78	2.66	2.66

^*^p < 0.05, ^**^p < 0.01; smallest profile (%)-the proportion of the category with the smallest population in the total sample.

**Table 3:** Classification probabilities for the four-profile model.

Profiles	1	2	3	4
1	**0.99**	0.06	0.01	0.00
2	0.00	**0.96**	0.03	0.01
3	0.01	0.02	**0.97**	0.00
4	0.00	0.01	0.00	**0.99**

Values indicate probabilities of most likely class membership (column) by latent class modal assignment (row). Bolded values indicate average posterior probabilities.

**Table 4:** Estimated means and standard deviations for the four-profile model (*N* = 866).

Profiles	Profile 1: Moderate mixed emotions (*n* = 220)	Profile 2: Blends of high positive emotions (*n* = 250)	Profile 3: Blends of moderate positive emotions (*n* = 268)	Profile 4: High mixed emotions (*n* = 128)
Indicators	M ± SD	M ± SD	M ± SD	M ± SD
Enjoyment	3.35 ± 0.47	4.37 ± 0.54	3.94 ± 0.40	4.17 ± 0.46
Hope	3.31 ± 0.48	4.34 ± 0.56	3.91 ± 0.47	4.18 ± 0.45
Anger	3.01 ± 0.39	1.26 ± 0.38	2.21 ± 0.47	4.10 ± 0.52
Boredom	3.06 ± 0.28	1.04 ± 0.13	2.05 ± 0.26	4.16 ± 0.44

**Figure 2:**
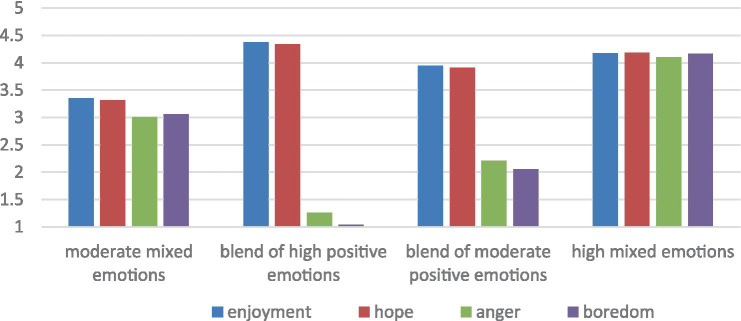
Four-profile solution of AEs with raw mean scores.

Table 4 and Figure 2 provide details on the characteristics of each profile. We labeled the most prominent emotion for a profile as high or low if it was closer to the endpoint than the midpoint of the scale (e.g., above 4 or below 2 on a 5-point scale; Wormington and Linnenbrink-Garcia, 2017). Profile 1 included 25.40% of the sample (*n* = 220) and was named “moderate mixed emotions” due to moderate mean scores on both positive and negative emotions. Profile 2 included 28.87% of the sample (*n* = 250) and was labeled “blends of high positive emotions” due to high mean scores on positive emotions. Profile 3 comprised 30.95% of the sample (*n* = 268) and was labeled “blends of moderate positive emotions” due to moderate mean scores on positive emotions and nearly two on negative emotions. Profile 4 comprised 14.78% of the sample (*n* = 128) and was labeled “high mixed emotions” due to high mean scores on both positive and negative emotions.

### 3.3 Predictors of achievement emotion profiles

We used the added predictive variable (the R3STEP approach) to explore the predictors of achievement emotion profiles (Table 5). In the analysis, the moderate mixed emotion profile was used as a reference.

**Table 5:** Predictive factors of achievement emotion profiles.

Predictors	Blends of high positive emotion profile β (95% CI)	Bends of moderate positive emotion profile β (95% CI)	High mixed emotion profile β (95% CI)
Gender	1.11(0.67–1.84)	1.43(0.93–2.18)	0.62(0.40–0.98)
Prior achievement	1.56(1.17–2.08)	1.30(1.05–1.60)	1.11(0.87–1.42)
Teacher support	168.75(67.99–418.85)	22.23(10.07–49.01)	0.70(0.26–1.90)

The moderate mixed emotion profile as the reference profile; OR, odd ratio; CI, confidence interval.

The results showed that there was no gender difference between individuals in the blends of high positive emotion or the blends of moderate positive emotion profile compared to those in the moderate mixed emotion profile. However, the proportion of females in the high mixed emotion profile was 38% higher than that of males. In summary, compared to males, females were more likely to belong to the high mixed emotion profile.

The results indicated that individuals with better prior achievement were 1.56 times more likely to belong to the blends of high positive emotion profile than to the moderate mixed emotion profile. Similarly, individuals with better prior achievement were 1.3 times more likely to belong to the blends of moderate positive emotion profile than to the moderate mixed emotion profile. In other words, students with higher prior achievement were more likely to be classified into the blends of positive emotion profile.

Furthermore, the results demonstrated that individuals who received higher teacher support were 168.75 times more likely to belong to the blends of high positive emotion profile than to the moderate mixed emotion profile. Similarly, individuals who received higher teacher support were 22.23 times more likely to belong to the blends of moderate positive emotion profile than to the moderate mixed emotion profile. In other words, more teacher support predicted a higher likelihood of being classified into the blends of positive emotion profile relative to the moderate mixed emotion profile.

### 3.4 The mediation

Direct and indirect effects are shown in Table 6. The results showed that the indirect effects of teacher support on achievement emotion profiles through need satisfaction were significant. These findings emphasized the mediating role of need satisfaction in the relationship between teacher support and the co-occurrence of students’ achievement emotions.

First, students who received more support from their teachers were more likely to experience need satisfaction, making them less likely to fall into the moderate mixed emotion profile. The indirect relationship between teacher support and the blends of moderate positive emotion profile was statistically significant (*β* = −0.12, *p* < 0.001). The direct relationship between teacher support and emotion profile also remained statistically significant (*β* = −0.21, *p* < 0.001).

**Table 6:** Adjusted direct indirect and total associations of teacher support with achievement emotion profiles via need satisfaction.

Achievement emotion profiles	β (95% CI)	*p* value
Moderate mixed emotion profile		
Direct Effect	−0.21(−0.27 to-0.16)	<0.001
Indirect Effect	−0.12(−0.15 to −0.09)	<0.001
Total Effect	−0.33 (−0.38 to-0.28)	<0.001
Blends of high positive emotion profile		
Direct Effect	0.47 (0.42–0.53)	<0.001
Indirect Effect	0.03(0.002–0.050)	0.033
Total Effect	0.50 (0.45–0.55)	<0.001
Blends of moderate positive emotion profile profiles		
Direct Effect	0.11(0.04–0.18)	<0.001
Indirect Effect	−0.06 (−0.09 to −0.03)	0.001
Total Effect	0.05(−0.01-0.11)	0.128
High mixed emotion profile		
Direct Effect	−0.37(−0.41 to −0.32)	<0.001
Indirect Effect	0.15(0.13–0.18)	<0.001
Total Effect	−0.22(−0.26 to-0.17)	<0.001

Second, students who received more support from their teachers were more likely to experience need satisfaction. In turn, this made them more likely to belong to the blends of high positive emotion profile. The indirect relationship between teacher support and the blends of high positive emotion profile through need satisfaction was statistically significant (*β* = 0.03, *p* < 0.05). The direct relations of teacher support on emotion profile also remained statistically significant (*β* = 0.47, *p* < 0.001).

Third, students with more teacher support were more likely to experience need satisfaction, leading to a decreased likelihood of belonging to the blends of moderate positive emotions. The indirect relationship between teacher support and the high mixed emotion profile through need satisfaction was significant (*β* = −0.06, *p* = 0.001). The direct relationship between teacher support and this emotion profile also remained statistically significant (*β* = 0.11, *p* < 0.001).

Finally, students who received more support from their teachers experienced more need satisfaction, making them more likely to fall into the high mixed emotion profile. The indirect relations between teacher support and the high mixed emotion profile through need satisfaction were statistically significant (*β* = 0.15, *p* < 0.001). The direct relations between teacher support and this emotion profile also remained statistically significant (*β* = −0.37, *p* < 0.001).

## 4 Discussion

We explored the co-occurrence of achievement emotions among college students and examined the impact of teacher support on achievement emotion profiles, as well as the mediating effect of need satisfaction. The present study found that four emotion profiles were identified. Notably, higher perceived levels of teacher support were associated with a greater likelihood of students being classified into the blends of positive emotion profile. Furthermore, the relationship between teacher support and all emotion profiles was mediated by the satisfaction of basic psychological needs.

### 4.1 How many emotion profiles would exist among Chinese college students?

Regarding RQ1, this study primarily focuses on the general achievement emotion profiles of college students. These findings support H₁. The emotional experiences of college students during daily learning were classified into four homogeneous subgroups: moderate mixed emotion profile, blends of moderate positive emotion profile, blends of high positive emotion profile, and high mixed emotion profile. The results further confirm the existence of co-occurring emotions and partially validate the two-level hierarchical structure of emotions proposed by Watson and Stanton (2017). Similarly, the study emphasizes that the same stimulus or event could induce multiple emotions, which are blended into different profiles for different people (Hu et al., 2022).

Among college students, a significant proportion (moderate positive 30.95%, high positive 28.87%) belonged to the blends of positive emotion profile, indicating that they were predominantly influenced by positive emotions during learning. The result was consistent with previous research (Ganotice et al., 2016; Robinson et al., 2017; Earl et al., 2024). In this study, it was implied that students experienced more enjoyment and hope during daily learning. The emotion patterns dominated by these positive emotions would likely predict higher attainment and attendance (Earl et al., 2024). However, it was not as expected; the blends of negative emotion profile did not appear. In other words, Chinese university students experienced less anger and boredom. A study found that, due to the impact of the pandemic, college students were forced to take online classes, which led to them experiencing more negative emotions during learning (Yuan, 2023). However, the study did not include the blends of negative achievement emotion profile. We speculate that this is due to the improvement in the COVID-19 pandemic situation, leading to a gradual resumption of face-to-face learning. All participants in this study attended school and actively participated in offline learning. It is common knowledge that achievement emotions vary across learning situations and contexts (Pekrun and Perry, 2014). During offline classes, students have more opportunities for interactions with the instructor and other learners, which can lead to a more positive attitude and a more enjoyable learning environment. As a result, students tend to experience more positive emotions (Frenzel et al., 2018; Yu et al., 2020). Therefore, students were less likely to have the negative emotion profile. However, even with the return to face-to-face learning, daily teaching practices may still undergo changes due to the pandemic, creating an environment fraught with uncertainty. The results of this study showed that over 40% of students experience mixed emotions. The mixed emotions experienced by students can indeed arise from conflicts caused by various changes, aligning with the conflict perspective of mixed emotions (Larsen, 2017; Oh, 2022). Additionally, the higher proportion of mixed emotions may be attributed to cultural differences between the East and West. Research has shown that East Asians tend to be more attuned to dialectical emotions, which involve experiencing seemingly contradictory emotions simultaneously (Miyamoto et al., 2010; Grossmann and Ellsworth, 2017). As previous research has indicated, the impact of mixed emotions on students’ academic performance may exhibit dual aspects (Robinson et al., 2017; Tze et al., 2020), so teachers need to pay more attention to students in this emotional profile.

### 4.2 How would antecedents affect students’ achievement emotion profiles?

In response to RQ2, an analysis was conducted to identify factors that significantly predicted achievement emotion profiles. The results partially support H₂.

The results showed that, when taking the moderate mixed emotion profile as the reference, gender differences only existed in the high mixed emotion profile. This finding aligns with the gender differences observed in emotional experiences reported among college students engaged in engineering tasks (Chen et al., 2023). In previous studies, gender differences were mainly reflected in specific learning areas, such as women excelling in language and men excelling in math or technology (Hirnstein et al., 2023), which could impact their beliefs about their abilities and subsequently affect their particular achievement emotions. The study sheds light on how gender influences emotion profiles in daily learning. The results indicated that female college students were more likely to belong to the high mixed emotion profile. According to the conflict perspective of mixed emotions, mixed emotions represent a state of conflict and agony (Durso et al., 2016; Larsen, 2017; Mejía and Hooker, 2017). Therefore, when individuals face conflicting goals, they are more likely to experience mixed emotions (Larsen, 2017). Similar studies have also found that female college students feel more pressure during learning; thus, they are more likely to experience burnout (Asikainen et al., 2022). Given the significant emphasis on academic performance in college, this pressure could exacerbate the conflicts they face, resulting in a greater likelihood of experiencing mixed emotions (Oh, 2022). Experiencing mixed emotions for long periods in non-crisis contexts can impair individual psychological well-being and social functioning (Oh, 2022; Oh and Tong, 2022). Therefore, it is important for educators to pay attention to the achievement emotions of female college students.

The findings also suggested that individuals with higher prior achievement were more likely to exhibit a positive emotion profile, which is in line with previous research (Karamarkovich and Rutherford, 2021). The results again confirm the importance of prior achievement as a significant factor in shaping students’ emotion profiles, consistent with variable-centered studies (Pekrun et al., 2017, 2023). Grades, serving as a form of feedback, positively predicted positive emotions and inversely predicted negative emotions over a span of 5 years (Pekrun et al., 2017, 2023). In summary, it is evident that prior academic performance influences both discrete achievement emotions and achievement emotion profiles. Prior research has revealed that students’ achievement emotion profiles tend to remain stable over a certain period (Tze et al., 2020). This also highlights the importance of educators developing effective strategies when providing feedback. Prior academic performance affects individual achievement emotion profiles, but it has limited value in assessing individual improvement and does not provide guidance on how to enhance future learning. In addition to providing simple grading feedback, there is also a need for targeted guidance or tailored teacher support.

Furthermore, teacher support was found to be a significant predictor of students’ emotion profiles. Compared with a moderate mixed emotion profile, higher perceived teacher support increased the likelihood of students belonging to the blends of positive emotion profile. This result highlights the significance of teacher support in influencing students’ co-occurrence of emotional experiences and reaffirms the importance of a supportive environment (Ryan and Deci, 2017). Specifically, an environment that supports autonomy and fosters competence can result in optimal growth and development for individuals.

In a supportive learning environment, teachers are able to consider students’ perspectives, act in ways that encourage choice and self-initiation, and provide meaningful rationales and relevance (Reeve, 2016). This approach can lead to stronger learning motivation (Coudevylle et al., 2020) and more positive emotions (Liu et al., 2021). Providing students with a supportive environment can also lead to the blends of positive emotion profile, which enhances learning motivation and contributes to academic achievement (Ganotice et al., 2016; Tze et al., 2020; Earl et al., 2024).

Additionally, the activation of brain regions varies across different emotion profiles, indicating that the co-occurring pattern of one’s emotional experiences may indeed reflect individual differences (Hu et al., 2022). Teacher support, as a crucial environmental factor for college students, plays a significant role in shaping students’ achievement emotion profiles. In this study, teacher support is a composite of three types of support rather than focusing on a specific type. This is consistent with previous research, which confirms that an optimal learning environment relies on the interconnectedness of teacher reliance on autonomy support, structure, and involvement (Olivier et al., 2021). This finding holds profound implications for higher education.

### 4.3 How does teacher support impact students’ achievement emotion profiles through need satisfaction?

In response to RQ3, a separate path analysis for each profile was performed. The results support H₃. Overall, the findings support the basic psychological needs theory (Vansteenkiste et al., 2020). By demonstrating the mediating effect of basic psychological needs, this study provides a deeper understanding that teacher support not only directly influences all emotion profiles but also through the fulfillment of students’ psychological need satisfaction.

Teacher support plays a role in cultivating the need satisfaction of students (Bureau et al., 2022). More need satisfaction can foster intrinsic motivation (Vansteenkiste et al., 2020), ultimately leading to a high level of positive emotions and a low level of negative emotions (Liu et al., 2021). Thus, students are more likely to belong to the blends of high positive emotion profile but are less likely to fall into the moderate mixed emotion profile and the blends of moderate positive emotion profile. In other words, the mediating effect of need satisfaction also had a significant impact on students’ achievement emotion profiles. It increased the likelihood of individuals entering a highly positive emotion profile while reducing the likelihood of individuals entering moderately positive and moderately mixed emotion profiles.

However, the mediating effect through need satisfaction increased the likelihood of students falling into the high mixed emotion profile. We speculate that this may be due to the multidimensional nature of the support provided by teachers and the diverse and complex needs of students (Olivier and Archambault, 2017). In addition to need satisfaction, there is also need frustration. These two constructs are emphasized as distinct constructs that can potentially coexist simultaneously (Warburton et al., 2020). It is possible that some support provided by teachers might be more effective in meeting students’ needs, and then students will experience more positive emotions. Conversely, when some support provided by teachers does not support students’ needs or even thwarts them, need frustration may increase, resulting in more negative emotions. When students experience simultaneous high emotions with different valences, they may subsequently be labeled as having a mixed emotion profile. Additionally, we analyzed the perception of teacher support and need satisfaction among individuals with a high mixed emotion profile and found students in this emotion profile had the lowest teacher support but the highest need satisfaction. The seemingly contradictory result highlights an intriguing finding. However, it is also quite understandable, considering that teacher support is just one facet contributing to meeting students’ needs. The overall need satisfaction of students is influenced by various contextual factors, such as teachers’ interpersonal behaviors (Behzadnia, 2021), teacher–student interactions (Bakadorova and Raufelder, 2018), and so on. High levels of need satisfaction lead to students experiencing more positive emotions. Furthermore, the results of this study also indicate that the direct effect of teacher support on achievement emotion patterns is greater than the mediating effect of need satisfaction (see Table 6). When teacher support is at its lowest, students are more likely to experience increased negative emotions (Lei et al., 2018). In comparison to positive emotions, the variability of negative emotions is a stronger predictor of mixed emotions (Barford et al., 2020). Therefore, when individuals experience an increase in negative emotions while positive emotions consistently remain moderate to high (see Table 4), they experience more mixed emotions. This is consistent with the notion that mixed emotions in daily life are predominantly influenced by fluctuations in negative emotions (Barford et al., 2020). In summary, the results of this study all support the mediating role of need satisfaction, emphasizing its particular significance in the influence of teacher support on the achievement emotion profiles of college students.

### 4.4 Limitations and direction for future research

This study identified four distinct patterns of achievement emotions among Chinese university students. Moreover, it expands upon BPNT by first adopting a person-centered perspective and examining the impact of teacher support on students’ achievement emotion profiles, as well as the mediating effect of need satisfaction. These findings carry valuable practical implications for higher education. However, the study also has some limitations. First, the cross-sectional survey design did not allow for causal inferences regarding relationships between teacher support, need satisfaction, and achievement emotion profiles. Longitudinal approaches are required to better understand how these predictive relationships develop and change over time. Second, the study only focused on four achievement emotions; other emotions, such as anxiety, were not considered. Previous research has indicated that variations in achievement emotions may yield differing results, thereby potentially imposing limitations on result interpretation. Future studies can encompass a more diverse array of achievement emotions. Finally, the study exclusively examined the influence of teacher support on the co-occurrence of students’ achievement emotions. The findings suggest that teacher support not only directly affects the co-occurrence of achievement emotions but may also exert a mediation through the satisfaction of students’ needs. This extends beyond previous research, which has been constrained to discrete achievement emotions. However, this study did not delve into how the co-occurrence of achievement emotions impacted students’ learning outcomes. A study found that achievement emotion profiles mediated the relationship between math value and achievement during online mathematics learning for fourth and fifth-grade elementary school students (Karamarkovich and Rutherford, 2021). Nonetheless, further verification is required to determine the applicability of these findings to students across different grade levels. Additionally, the impact of achievement emotion profiles across learning styles, such as offline or gamified learning, is a question worth exploring.

### 4.5 Educational implications

This study marks an attempt to extend the influence of teacher support into the realm of the co-occurrence of emotions, reaffirming the significant role of teachers in shaping individuals’ diverse emotion profiles and offering valuable educational insights. First, it is evident that students experience different achievement emotions during the learning process. Therefore, personalized support and guidance from teachers are crucial for students with different achievement emotion profiles. Second, establishing a supportive learning environment is crucial. Teacher support could help students cultivate positive achievement emotion profiles, which in turn could enhance the sense of engagement in learning and lead to better academic performance (Robinson et al., 2017; Karamarkovich and Rutherford, 2021). Thus, educators should strive to create a supportive learning atmosphere to foster student development. Finally, the mediation of need satisfaction highlights the relationship between teacher support and achievement emotion profiles. This highlights the significance of teachers simultaneously addressing students’ autonomy, competence, and relatedness needs while providing support, as the global level of support offered by teachers exerts the most substantial influence on classroom engagement (Olivier et al., 2021).

## 5 Conclusion

Our study is the first to examine the membership of emotion profiles within a BPNT framework among Chinese college students. Four emotion profiles in the daily achievement emotions were found in college students: moderate mixed emotion profile, the blends of high positive emotion profile, the blends of moderate positive emotion profile, and high mixed emotion profile. In addition, teacher support could significantly predict students’ emotion profiles in most cases. Need satisfaction played a mediating role in the association between teacher support and four profiles.

## Data availability statement

The datasets generated and analyzed during the current study are available from the corresponding author on reasonable request. Requests to access these datasets should be directed to gongsy@ccnu.edu.cn.

## Ethics statement

The study was approved by Central China Normal University, Ethic Committee. The studies were conducted in accordance with the local legislation and institutional requirements. Written informed consent to participate in this study was provided by the participants.

## Author contributions

YY: Data curation, Funding acquisition, Methodology, Writing – original draft, Writing – review & editing. SG: Conceptualization, Funding acquisition, Supervision, Writing – review & editing. YC: Supervision, Writing – review & editing. YQ: Formal analysis, Resources, Validation, Writing – review & editing. XX: Data curation, Methodology, Writing – review & editing. YW: Conceptualization, Writing – review & editing.
